# Counting White Blood Cells from a Blood Smear Using Fourier Ptychographic Microscopy

**DOI:** 10.1371/journal.pone.0133489

**Published:** 2015-07-17

**Authors:** Jaebum Chung, Xiaoze Ou, Rajan P. Kulkarni, Changhuei Yang

**Affiliations:** 1 Department of Electrical Engineering, California Institute of Technology, Pasadena, California, United States of America; 2 Division of Dermatology, University of California Los Angeles, Los Angeles, California, United States of America; 3 Department of Bioengineering, California Institute of Technology, Pasadena, California, United States of America; University of California San Diego, UNITED STATES

## Abstract

White blood cell (WBC) count is a valuable metric for assisting with diagnosis or prognosis of various diseases such as coronary heart disease, type 2 diabetes, or infection. Counting WBCs can be done either manually or automatically. Automatic methods are capable of counting a large number of cells to give a statistically more accurate reading of the WBC count of a sample, but the specialized equipment tends to be expensive. Manual methods are inexpensive since they only involve a conventional light microscope setup. However, it is more laborious and error-prone because the small field-of-view (FOV) of the microscope necessitates mechanical scanning of a specimen for counting an adequate number of WBCs. Here, we investigate the use of Fourier ptychographic microscopy (FPM) to bypass these issues of the manual methods. With a 2x objective, FPM can provide a FOV of 120 mm^2^ with enhanced resolution comparable to that of a 20x objective, which is adequate for non-differentially counting WBCs in just one FOV. A specialist was able to count the WBCs in FPM images with 100% accuracy compared to the count as determined from conventional microscope images. An automatic counting algorithm was also developed to identify WBCs from FPM’s captured images with 95% accuracy, paving the way for a cost-effective WBC counting setup with the advantages of both the automatic and manual counting methods.

## Introduction

White blood cells are the effector cells of the immune system and circulate throughout the bloodstream and lymphatic system. An infection or a physical injury results in an inflammatory response, which induces increased production of WBCs for resolving the injury or infection. Due to this association between WBCs and inflammatory response, WBC count is a valuable metric for diagnosis and prognosis of several diseases. Several researchers have found that a high WBC count is strongly related to the risk of coronary heart disease [1–6], which further elucidated the link between the cardiovascular disease and inflammation [4]. Smoking was found to cause an increase in WBC count, but there is growing evidence that a high WBC count is a predictor of coronary heart disease regardless of one’s smoking status [5]. Not only does the high WBC count predict the development of macro- and micro-vascular complications in patients with type 2 diabetes [7], but the future onset of type 2 diabetes has also been linked to a high WBC count [8]. In pediatrics, a high WBC count has been found to be an indicator of bacteremic infection in children [9]. Thus, WBC count can be a valuable tool for prognosis of diseases.

In the broadest sense, there are two ways to count WBCs: an automatic method and a manual method. Flow cytometry is a common automatic method that works by flowing WBCs in a single file through electronic detectors [10]. The detectors then quantify the optical and electrical properties of the stream of fluid to differentially count the five types of WBCs. This method is convenient for analyzing large volumes of blood samples in the most time efficient manner, and is becoming more economical with advances in microfluidics flow cytometry [11]. However, because it does not capture any images of the cells being analyzed, further investigation into the cells’ morphology and variation is almost impossible. Moreover, it cannot identify cells with abnormal physiology because its identification method depends on the accepted ranges for the signature characteristics of the cells in the blood. Image cytometry bypasses these problems by also capturing the images of the flowing analytes on top of performing flow cytometry [12]. Although it is very powerful, it is unfavorable for resource-limited research labs or clinics due to its expensive price tag.

A manual counting method is an alternate way to count WBCs, but with much lower throughput. The manual WBC counting method can be performed on either a blood smear sample or a hemocytometer using a standard microscope system. The specimen can either be viewed directly through the microscope’s eyepiece or captured into image files. For a blood smear sample, the monolayer regions are mechanically scanned for counting the total number of WBCs [13], while for a hemocytometer, the gridded area is scanned for the counting purpose [14]. Although the manual method is more laborious and time consuming, it offers scientists the flexibility to use a wide array of objective lenses available for the standard microscope for careful visual analysis of the specimens. High-quality yet inexpensive imaging devices of today’s technology allow for this application in resource-limited settings [15].

One of the technical problems associated with the manual counting method is the error associated with the mechanical scanning of the glass slide. A conventional microscope’s performance is limited by its space-bandwidth product, meaning that there is a trade-off between the image’s resolution and the microscope’s field-of-view (FOV). Generally, in order to view and non-differentially count WBCs under a conventional microscope, an objective with the magnification power of at least 10x (0.25NA) is used [16]. For a differential WBC count, an oil-immersion objective with around 100x magnification (1.4NA) is used. However, at these high magnification powers, the FOV is very small, which necessitates mechanical scanning of the glass slide during the counting process. This is unfavorable because the scanning movement has to be precisely aligned and controlled to avoid any overlap of the scanning regions. Another disadvantage of using standard microscopy for manual counting is the physical strain on clinicians associated with the manual scanning of the slide and direct observation through the microscope. Routine performance of analyzing specimens under a standard microscope may be detrimental to the clinicians [17].

Here, we investigate the use of Fourier Ptychographic Microscopy (FPM) as a solution that can remedy the issues inherent in the manual WBC counting method. FPM is a novel microscope imaging method first reported in [18] that can computationally stitch together a series of lower resolution, wide FOV images in the Fourier domain to produce a higher resolution, wide FOV image. With FPM, large area of the monolayer region of a blood smear can be imaged free of any mechanical movement involved in scanning. FPM requires minimal modifications to a conventional microscopy setup, only involving an LED matrix and a CCD camera. We demonstrate its viability for use in counting WBC by having a specialist in hemocytometry compare its performance against that of a conventional 20x microscope setup, which acts as the ground truth in our study. Only non-differential WBC count was conducted with the setup described in the following sections. FPM’s potential for differential WBC count is noted. Finally, the effectiveness of an automatic WBC counting algorithm on the FPM images is discussed.

## Principles of FPM

**Fig 1:**
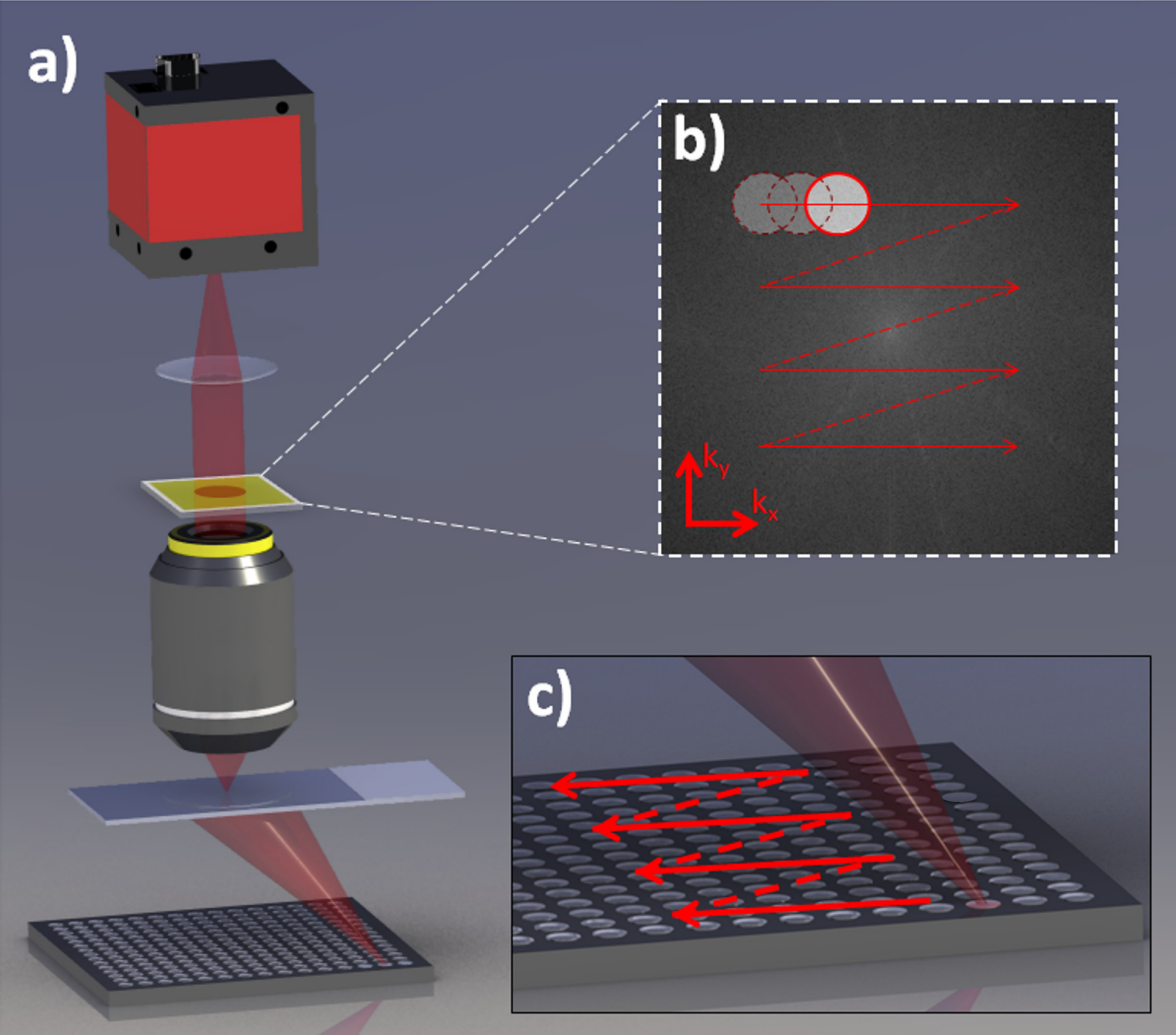
FPM setup. a) The schematics of FPM setup. It consists of a conventional microscope, involving an objective, tube lens, a camera, and an LED matrix replacing the condenser for specimen illumination. b) Fourier spectrum of the sample at the objective’s back-focal plane. The circular subregion corresponds to the objective’s aperture size. It shifts with the shifting illumination angle. c) Shifting illumination angle is provided by illuminating LEDs at different locations on the matrix.

FPM is a computational method that can effectively increase the NA of a microscope system to acquire a high-resolution and complex-amplitude image of the specimen with the benefit of the wide FOV associated with a low NA objective. The technology makes use of two key elements: (1) the fact that illuminating a specimen with an oblique plane wave in a microscope setup results in laterally shifting the specimen’s Fourier spectrum in the back focal plane of the objective lens; and (2) the ability to recover the phase information of the specimen from its intensity image by a phase retrieval algorithm [18]. FPM setup, as shown in Fig 1, involves a conventional microscope setup with its light source replaced by an LED matrix. We assume our LEDs to be quasi-monochromatic, and the more detailed analysis is found in [19]. The light field emitted from one LED can be approximated as a plane wave within a small region at the sample plane because the large distance (~8 cm) between the LED and the specimen increases the spatial coherence of the LED. The plane wave has vector components (kx, ky) associated with the oblique illumination of the LED on the sample. Lighting up one LED causes the sample’s Fourier spectrum to be shifted by (kx, ky) at the objective lens’s back-focal plane, and the finite numerical aperture (NA) of the objective lens acts as a low-pass filter that transmits only a small subregion at the center of the shifted Fourier spectrum. The low-passed Fourier component is further propagated to the image plane and is captured by a camera. LEDs are lit up sequentially so that the captured images contain partially overlapping Fourier subregions that together span the entire Fourier spectrum of the specimen. The captured images are broken up into smaller tiles of spatially coherent regions, whose dimension is given by the van Cittert-Zernike theorem: *L* = 0.61*λz/a* [20], where *λ* is the LED wavelength, *z* is the LED to sample distance, and *a* is the radius of the LED’s active area. With the LED dimension of 200 μm x 200 μm, distance between the LED matrix and the sample of 8 cm, and the wavelength of 630 nm, the coherence length is ~307 μm. Also within this dimension, the spatially varying aberrations are assumed to be constant. We reconstruct each tile separately so that the spatially varying aberration across the microscope’s FOV can be addressed by the reconstruction algorithm discussed below.

For each tile in the FOV, it is possible to recover its expanded complex Fourier spectrum from the variably illuminated intensity images by applying the phase retrieval algorithm reported in [21], called embedded pupil function recovery (EPRY) algorithm. The algorithm iteratively solves for the expanded Fourier spectrum by using the intensity images as a constraint in the spatial domain and the objective’s finite NA as a shifting low pass constraint in the Fourier domain. In this process, the algorithm is able to not only generate the high-resolution complex amplitude image of the specimen, but also separate the pupil function and the specimen’s complex amplitude function from the image. The pupil function contains the aberrations of the microscope associated with its lens system and the defocus caused by misalignment of the sample at the focal plane, both of which can be dependent on the tile’s location in the FOV. Therefore, the specimen’s complex amplitude function is an aberration-corrected, diffraction-limited complex image of the specimen. By stitching the tiles together in the spatial domain, we obtain the specimen’s intensity image and phase information separated from aberrations across the entire FOV.

## Materials and Methods

### Ethics Statement

The study was approved by the Caltech Committee for the Protection of Human Subjects (IRB) at The California Institute of Technology (IRB No. 14–0464). All the blood specimens used in this research project were procured after obtaining a signed consent form from the volunteers. No volunteers are identified with the specimens in this project.

### Blood Smear Preparation

A Wright-Giemsa stained blood smear was prepared for testing FPM’s performance against that of the conventional microscopy. 2.0 mg EDTA/mL was added to a whole blood sample, and 1 μL of the mixture was smeared uniformly across a cover glass using another cover glass at a 30 degree smearing angle. After the specimen was air-dried for 5 minutes, HEMA 3 Wright-Giemsa staining kit from Fisher Diagnostics was used to fix and stain the specimen. The kit involved dipping the specimen for 1 second sequentially into three Coplin jars, with the first one containing methanol-based HEMA 3 fixative solution, the second containing HEMA 3 solution I, and the last containing HEMA 3 solution II. The dipping steps were repeated 5 times for each specimen. Then, the specimen was rinsed with deionized water and air-dried for another 5 minutes.

### Imaging using FPM and Conventional Microscopy Method

An Olympus BX 41 microscope with 20x objective (UPlanFL N, 0.5 NA, Olympus) was used for the conventional WBC counting method. 20 different areas on the blood smear specimen were imaged using a CMOS color camera (MT9P031, 2.2μm pixel size). For FPM method, the same microscope with a 2x objective (PlanApo N, 0.08 NA, Olympus) was used with red LEDs (SMD 3528, 632nm central wavelength, ~10nm bandwidth after a bandpass filter) as the light source, and the same 20 regions were extracted from its wide FOV image captured by an inline CCD camera (Kodak KAI-29050, 5.5-mm pixel size). 15 x 15 LEDs were sequentially lit up for the capturing process, providing an effective NA of ~0.5 for the FPM system. The entire capturing process took ~3 minutes (limited by the LEDs’ brightness and the camera’s frame-rate) and the reconstruction ~10 minutes (limited by the computer’s processing speed). We used monochromatic images for this study because only the contrast information was needed for identifying WBCs from the specimen.

### Blinded counting of WBCs

The images of the 20 blood smear regions obtained by the conventional 20x microscope system were analyzed by a trained specialist. The WBC count for each image was tabulated, establishing the baseline value for testing the FPM’s performance. To ensure that the study was blinded, the specialist was given a 2-week time window before he analyzed the corresponding 20 regions obtained by FPM. Moreover, the order of the FPM images was randomized. The WBC count for FPM images was tabulated, and the 20 pairs of FPM and conventional microscope images were matched for comparing the respective systems’ WBC count in each region.

## Results and Discussion

**Fig 2:**
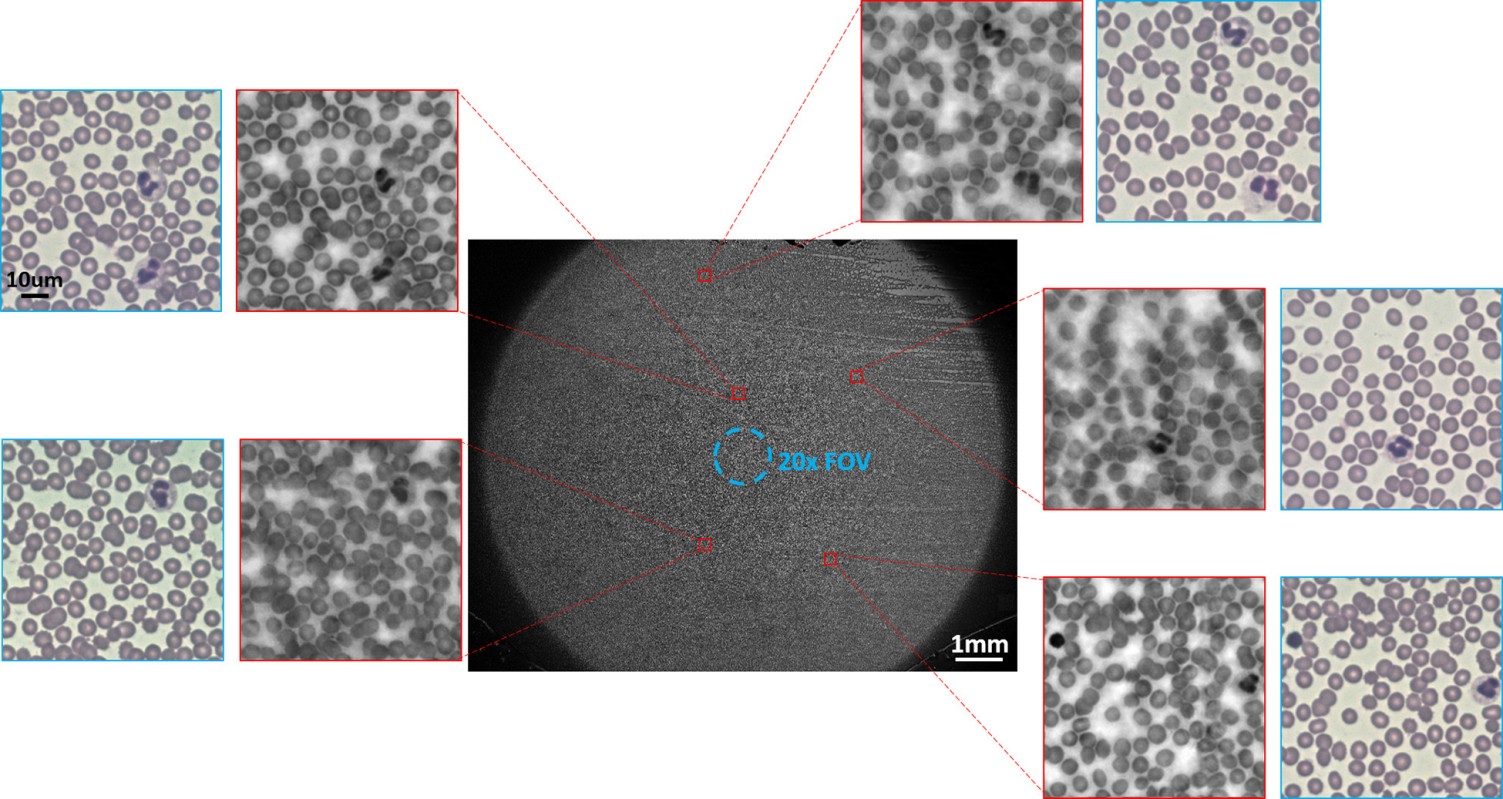
The full FOV image of a blood smear slide with SBP of ~0.9 gigapixel by FPM using 2x 0.08 NA objective (center) and its magnified subregions (surrounding). The red subregions are directly extracted from the full FOV image, and the corresponding blue subregions are taken by a 20x 0.5 NA objective. They both offer enough details from which we can discern the shapes and sizes of the WBCs and their nuclei. The blue circle in the middle of the center image represents the relative size of the FOV achieved by a 20x objective compared to that of FPM.

Fig 2 shows a comparison between the images of several regions acquired by the two methods. The FOV provided by FPM corresponds to about 120 mm^2^ in the sample plane, with the effective NA of 0.5 and full pitch resolution of 1560 nm, producing a ~1 gigapixel image. The FOV is significantly greater than what a 20x microscope can provide, which is about 1.2 mm^2^. Comparing windowed regions from FPM and the 20x conventional microscopy, we can clearly see the nuclei of WBCs in both images. The nuclei are, in fact, primarily used to distinguish the WBCs from the blood smear because WBCs are the only cells in the blood with a nucleus.

In the blinded comparison study, the specialist was able to count the same number of WBCs in each region for both conventional microscopy images and the FPM images. In total, 38 WBCs were counted for both methods, and there were no false positive or false negative readings from the FPM. The result is summarized in Table 1. This indicates that the images captured by FPM are of adequate, if not exceeding, quality for WBC counting procedure and are able to produce accurate diagnoses. Thus, we confirm that FPM is a suitable substitute for standard microscopy that hematologists can use to perform WBC count.

**Table 1:** WBC counting performance on 20 different regions on a blood smear slide using the 3 respective methods.

Method	Accuracy (%)	False Positive Rate (%)	False Negative Rate (%)
**Conventional**	100	0	0
**FPM**	100	0	0
**FPM + Automatic**	95	5.26	0

There were 38 WBCs in total. Conventional microscopy method was considered as the ground truth. A specialist could count WBCs using FPM with 100% accuracy. WBC count using a computer algorithm was accurate 95% of the time.

The key advantage of FPM, as demonstrated in Fig 2, is its ability to resolve a high-resolution image with a wide FOV. In this setup, we were able to acquire the blood smear image with the FOV provided by the 2x 0.08 NA objective with the resolution comparable to 20x 0.5 NA objective. The wide FOV is highly favorable for counting WBCs using a microscope because it eliminates the amount of mechanical scans a specialist would need to perform on the blood smear specimen to count enough WBCs for analysis. Manual mechanical scanning can introduce errors in the counting process by creating unintended overlaps between the scanning regions instead of imaging perfectly contiguous regions. However, with FPM, such errors are significantly reduced by lowering the number of required scanned regions, or are even eliminated when enough WBCs can be counted in one FOV. Considering that about ten 40x microscope fields are typically required for sufficiently estimating the total WBC count in blood, the total FOV required is ~5 mm^2^ [22]. FPM exceedingly satisfies this requirement with its 2x objective, which has a FOV of 120 mm^2^. In comparison, the 20x 0.5 NA objective has 1.2 mm^2^ FOV, which necessitates lateral scanning of the slide.

It is worth contrasting the FPM method with other whole slide imaging (WSI) methods that incorporate precisely aligned mechanical components to achieve a high-resolution, wide FOV image. There are several advantages that FPM has over these methods. One is the stability of the FPM due to the lack of any moving parts. The absence of moving parts implies less wear and tear, so the system’s integrity can be preserved for a longer period of time. Second, and arguably the biggest advantage of FPM, is FPM’s robustness to possible errors resulting from an improperly focused setup. In a standard WSI method, most image-quality problems are focus related [17]. Out-of-focus images entail repeating a new set of image acquisition after properly focusing the objective. In the case with FPM, the strenuous repetition is unnecessary due to its refocusing ability [18]. Because the image that FPM reconstructs is a complex image containing both its amplitude and phase, it can be digitally propagated in the z-axis to its correct focal plane, providing an effective depth of focus of 300 μm [18]. As a result, FPM can image a specimen with uneven surface profile such that the entire area is in focus across the imaged FOV. Blood smear samples with spatially varying thickness levels, or even tilted specimens from misalignment can be successfully imaged using FPM. Third, FPM is much less expensive than WSI. WSI usually involves a precision mechanical stage that can cost US$100,000~150,000 apiece [17] whereas FPM only requires adding an inexpensive LED matrix (US$50) to the existing conventional microscope system.

**Fig 3:**
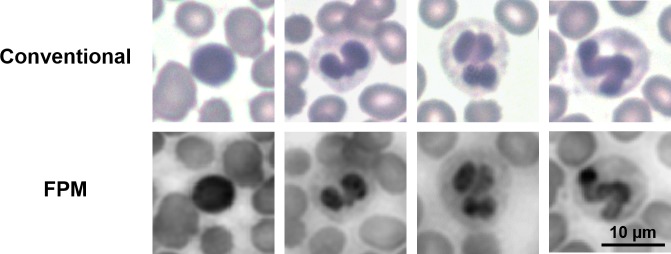
Several white blood cells from the imaged regions by a 20x conventional microscope (above) and FPM (below). Different morphologies can be observed among these cells. The left-most cell has an eccentric, single-lobed nucleus, suggesting that it is a lymphocyte, while other cells display multi-lobed nuclei structure, suggesting that they are eosinophils, basophils, or neutrophils.

The presence of nuclei in WBCs is identified from the FPM images. In addition, the nuclei’s morphology can be observed. Most of the WBCs in Fig 3 show the multi-lobed nucleus structure, which is an indicator for neutrophil, eosinophil, or basophil, and one WBC displays an eccentric nucleus, which is characteristic of a lymphocyte. In this study, differential WBC counting was not performed. However, the high-resolution FPM images suggest that using a higher NA objective and wider angles of LED illumination in FPM setup to further increase the effective NA up to ~1.4 may enable differential WBC counting on our system. For readers interested in the implementation of a high NA FPM system, please refer to [23]. Also, acquiring a full-color image of the blood smear may aid our system in performing differential counting because the color of the cells helps for distinguishing different types of WBCs.

**Fig 4:**
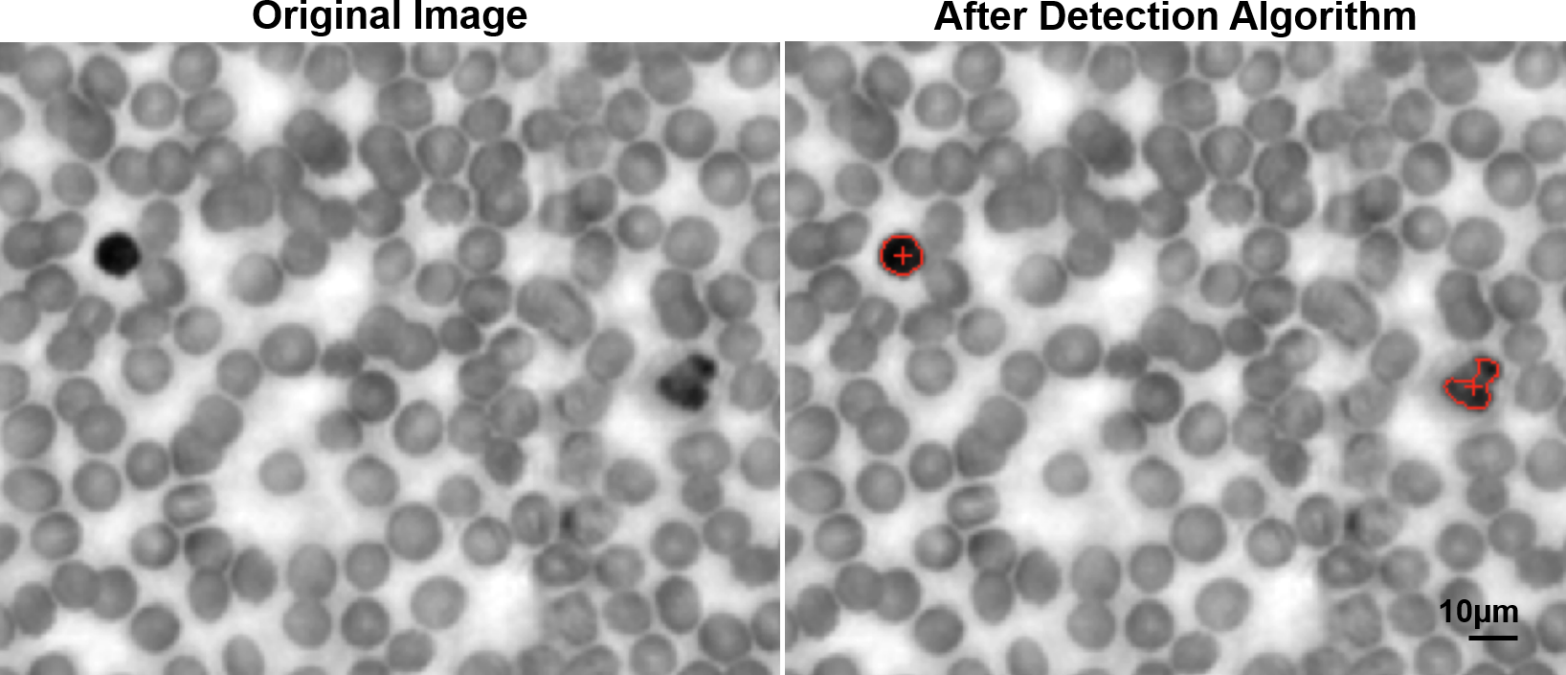
WBC detection by the automatic counting algorithm. The algorithm analyzes a section of the specimen image obtained by FPM (left), and outputs the image overlaid with red markings on the detected WBCs.

The clear morphological difference between the WBCs and RBCs in the smear images prompted us to apply an algorithm to automate the WBC counting process. Because of WBCs’ markedly high contrast originating from the presence of a nucleus, an algorithm could be developed to isolate the high contrast regions, and use them as the markers to identify the WBCs in the blood smear images. Using MATLAB, we quantified the difference in contrast and size of the WBCs compared to RBCs. With this information, the algorithm was able to identify WBCs with 95% accuracy across the 20 acquired sample images, as summarized in Table 1. An example of the algorithm’s WBC identification is shown in Fig 4. The errors in counting were found on images where (1) either the contrast of WBCs’ nuclei was not high against the background RBCs or (2) the RBCs were clumped up in one region, producing a contrast as high as that of WBCs’ nuclei in their proximity. Nevertheless, with the counting accuracy of 95%, the algorithm can assist clinicians in manually counting a large amount of blood samples. Including more parameters other than WBCs’ contrast and size in the program is expected to lead to higher accuracy.

## Conclusion

We have demonstrated that FPM can be useful for WBC counting thanks to its large FOV and high resolution that only require the addition of an inexpensive LED matrix to a conventional microscope setup. It significantly reduces or even eliminates the amount of scanning of the blood specimen required for manual counting of the WBCs. The low cost of FPM may enable a wider community to acquire high-resolution wide-field images comparable to those from WSI and to perform WBC count procedures more ergonomically. FPM’s digital acquisition of images also makes it a strong candidate for telemedicine applications. Further improvements in the image resolution will also enable differential WBC counting on FPM system. Applying more parameters for distinguishing WBCs from the blood sample to the automatic counting algorithm will lead to higher accuracy.

## References

[1] Yarnell JW, Baker IA, Sweetnam PM, Bainton D, O'Brien JR, Whitehead PJ, et al Fibrinogen, viscosity, and white blood cell count are major risk factors for ischemic heart disease. The Caerphilly and Speedwell collaborative heart disease studies. Circulation. 1991;83(3):836–44. 10.1161/01.cir.83.3.836 1999035

[2] Kannel WB, Anderson K, Wilson PF. White blood cell count and cardiovascular disease: Insights from the framingham study. JAMA. 1992;267(9):1253–6. 10.1001/jama.1992.03480090101035 1538564

[3] Grimm RH Jr, Neaton JD, Ludwig W. Prognostic importance of the white blood cell count for coronary, cancer, and all-cause mortality. JAMA. 1985;254(14):1932–7. 10.1001/jama.1985.03360140090031 4046122

[4] Barron HV, Cannon CP, Murphy SA, Braunwald E, Gibson CM. Association Between White Blood Cell Count, Epicardial Blood Flow, Myocardial Perfusion, and Clinical Outcomes in the Setting of Acute Myocardial Infarction: A Thrombolysis In Myocardial Infarction 10 Substudy. Circulation. 2000;102(19):2329–34. 10.1161/01.cir.102.19.2329 11067784

[5] Brown DW, Giles WH, Croft JB. White blood cell count: An independent predictor of coronary heart disease mortality among a national cohort. Journal of Clinical Epidemiology. 2001;54(3):316–22. 10.1016/S0895-4356(00)00296-1 11223329

[6] Twig G, Afek A, Shamiss A, Derazne E, Tzur D, Gordon B, et al White Blood Cell Count and the Risk for Coronary Artery Disease in Young Adults. PLoS ONE. 2012;7(10):e47183 10.1371/journal.pone.0047183 23077568 PMC3470580

[7] Tong PC, Lee K-F, So W-Y, Ng MH, Chan W-B, Lo MK, et al White blood cell count is associated with macro-and microvascular complications in Chinese patients with type 2 diabetes. Diabetes care. 2004;27(1):216–22. 14693992 10.2337/diacare.27.1.216

[8] Vozarova B, Weyer C, Lindsay RS, Pratley RE, Bogardus C, Tataranni PA. High White Blood Cell Count Is Associated With a Worsening of Insulin Sensitivity and Predicts the Development of Type 2 Diabetes. Diabetes. 2002;51(2):455–61. 10.2337/diabetes.51.2.455 11812755

[9] Jaffe DM, Fleisher GR. Temperature and Total White Blood Cell Count as Indicators of Bacteremia. Pediatrics. 1991;87(5):670–4. 2020512

[10] Shapiro HM. Practical Flow Cytometry. New Jersey: Wiley-Liss; 2003.

[11] Huh D, Gu W, Kamotani Y, Grotberg JB, Takayama S. Microfluidics for flow cytometric analysis of cells and particles. Physiological Measurement. 2005;26(3):R73 15798290 10.1088/0967-3334/26/3/R02

[12] Zuba-Surma EK, Kucia M, Abdel-Latif A, Lillard JW, Ratajczak MZ. The ImageStream System: a key step to a new era in imaging. Folia Histochemica et Cytobiologica. 2007;45(4):279–90. 18165167

[13] Kessel RG. Basic Medical Histology: The Biology of Cells, Tissues, and Organs. New York: Oxford University Press; 1998.

[14] Turgeon ML. Clinical Hematology: Theory and Procedures. Philadelphia: Lippincott Williams & Wilkins; 2005 551 p.

[15] Breslauer DN, Maamari RN, Switz NA, Lam WA, Fletcher DA. Mobile Phone Based Clinical Microscopy for Global Health Applications. PLoS ONE. 2009;4(7):e6320 10.1371/journal.pone.0006320 19623251 PMC2709430

[16] Bellwood B, Andrasik-Catton M. Veterinary Technician's Handbook of Laboratory Procedures. Ames: Wiley-Blackwell; 2013.

[17] Ghaznavi F, Evans A, Madabhushi A, Feldman M. Digital imaging in pathology: whole-slide imaging and beyond. Annual Review of Pathology: Mechanisms of Disease. 2013;8:331–59. 10.1146/annurev-pathol-011811-120902 23157334

[18] Zheng G, Horstmeyer R, Yang C. Wide-field, high-resolution Fourier ptychographic microscopy. Nat Photon. 2013;7(9):739–45. 10.1038/nphoton.2013.187 PMC4169052 25243016

[19] Horstmeyer R, Yang C. A phase space model of Fourier ptychographic microscopy. Opt Express. 2014;22(1):338–58. 10.1364/OE.22.000338 24514995 PMC3926543

[20] Mandel L, Wolf E. Optical Coherence and Quantum Optics. New York: Cambridge University Press; 1995.

[21] Ou X, Zheng G, Yang C. Embedded pupil function recovery for Fourier ptychographic microscopy. Opt Express. 2014;22(5):4960–72. 10.1364/OE.22.004960 24663835 PMC4086333

[22] Fritsma GA, Doig K, Rodak BF. Hematology: Clinical Principles and Applications. St. Louis: Elsevier Health Sciences; 2008.

[23] Ou X, Horstmeyer R, Zheng G, Yang C. High numerical aperture Fourier ptychography: principle, implementation and characterization. Opt Express. 2015;23(3):3472–91. 10.1364/OE.23.003472 25836203 PMC5802253

